# Continuous Flow Epoxidation of Alkenes Using a Homogeneous Manganese Catalyst with Peracetic Acid

**DOI:** 10.1021/acs.oprd.2c00222

**Published:** 2023-01-14

**Authors:** Ailbhe A. Ryan, Seán D. Dempsey, Megan Smyth, Karen Fahey, Thomas S. Moody, Scott Wharry, Paul Dingwall, David W. Rooney, Jillian M. Thompson, Peter C. Knipe, Mark J. Muldoon

**Affiliations:** † Almac Group, Craigavon BT63 5QD, United Kingdom; ‡ Arran Chemical Company, Roscommon N37 DN24, Ireland; § Queen’s University Belfast, Belfast BT9 5AG, United Kingdom

**Keywords:** flow chemistry, oxidation, catalysis, manganese, epoxidation, continuous processing

## Abstract

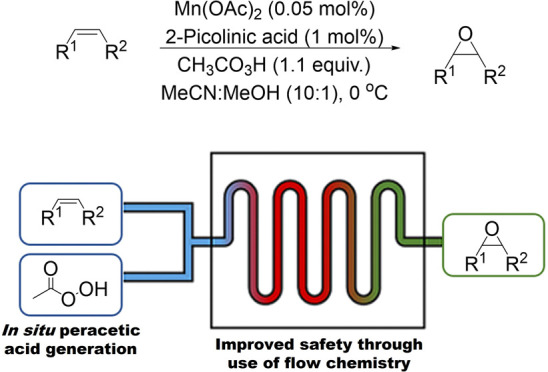

Epoxidation of alkenes is a valuable transformation in the synthesis of fine chemicals. Described herein are the design and development of a continuous flow process for carrying out the epoxidation of alkenes with a homogeneous manganese catalyst at metal loadings as low as 0.05 mol%. In this process, peracetic acid is generated *in situ* and telescoped directly into the epoxidation reaction, thus reducing the risks associated with its handling and storage, which often limit its use at scale. This flow process lessens the safety hazards associated with both the exothermicity of this epoxidation reaction and the use of the highly reactive peracetic acid. Controlling the speciation of manganese/2-picolinic acid mixtures by varying the ligand:manganese ratio was key to the success of the reaction. This continuous flow process offers an inexpensive, sustainable, and scalable route to epoxides.

## Introduction

Epoxides are a valuable class of compounds across the pharmaceutical and fragrance industries.^1,2^ They can be found in medicinal compounds such as carfilzomib (an anticancer agent) and troleandomycin (a macrolide antibiotic), though they are more often employed as key building blocks in the syntheses of fine chemical products.^3,4^ The reactivity of epoxides toward a broad range of nucleophiles makes them versatile intermediates.^5,6^

**Figure 1:**
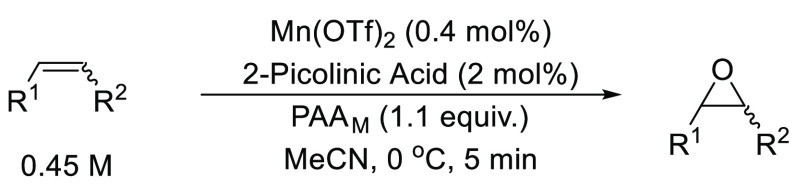
Alkene epoxidation using manganese triflate, 2-picolinic acid, and peracetic acid as reported by Stack and co-workers.^17^ PAA_M_ = 10:3:13 v/v/v 32 wt% PAA/10 wt% KOH(aq)/AcOH.

The oxidation of an alkene is an effective route for the preparation of epoxides; however, oxidations are often challenging due to the environmental and economic problems associated with traditional, stoichiometric oxidizing agents.^6^ For example, the storage, transport, and use of 3-chloroperoxybenzoic acid (mCPBA) on a large scale has been highlighted as a challenge for some time due to the risk of explosion.^7−9^ The use of catalysts with environmentally sustainable oxidants offers an attractive approach.^10^ Catalyst development is driven by a number of factors, with important drivers being substrate scope, product selectivity, and overall cost. The use of abundant, first-row transition metals is highly desirable, and therefore, many research groups interested in epoxidation reactions have reported catalysts based on manganese and iron.^11−13^ In the case of homogeneous catalysis, it is not only the choice of metal that is important but also the ligand. 2-Picolinic acid (pyridine-2-carboxylic acid) is a ligand that is both inexpensive and commercially available. Browne and co-workers previously employed 2-picolinic acid with manganese(II) salts and a hydrogen peroxide/butanedione oxidant for alkene, alcohol, and C–H oxidation reactions.^14−16^ Complementary work by Stack and co-workers used a similar catalyst system for alkene epoxidation but used a base-modified solution of commercial peracetic acid (PAA_M_) as the oxidant (Figure 1).^17^ This catalytic system facilitated the epoxidation of a broad range of alkenes, including terminal alkenes, at low catalyst loadings (0.4 mol%) and forms the basis of this study under flow conditions.

From an industrial perspective, the use of peracetic acid (PAA) is attractive, as it is an inexpensive oxidant which produces relatively benign byproducts and side products (acetic acid, oxygen, and water). Although PAA has been successfully utilized for very large scale applications such as wastewater treatment, its use in fine chemical synthesis is often hindered because of the associated risk of fire and explosion incidents due to the presence of metals or flammable organic chemicals or the use of heat.^18,19^ The catalytic method reported by Stack and co-workers demonstrated that alkenes could be converted to epoxides with very short reaction times. It was calculated that these epoxidation reactions are exothermic and exergonic, with Δ*H*_rxn_ = −40 to −50 kcal mol^–1^ and Δ*G*_rxn_ = −30 to −40 kcal mol^–1^.^20^ This suggests that they could be challenging to scale-up safely using batch reactors, as oxidations are known to pose a risk of thermal runaway and possible explosion.^21,22^ Utilization of a continuous flow process is regarded as the safest for the development of scalable oxidation protocols.^23−25^ The increased safety associated with flow systems is due to the smaller reactor volumes, which reduces the risks when using hazardous reagents.^26,27^ A smaller reactor volume is inherently safer, as it allows for greater control over the reaction conditions. Smaller reactor volumes also ensure that only a small fraction of the starting material or product is at risk (due to equipment failure or human error) at any one time, limiting the scale of any potential adverse event. Such process intensification not only means smaller reaction volumes but also superior heat transfer, something particularly important for this exothermic epoxidation. Exploiting continuous flow further, PAA can be prepared *in situ* and subsequently used for the desired reaction, which is an attractive option for accessing epoxidation.^28−30^

The design and development of a Mn(II)/2-picolinic acid catalyst system is described herein.

## Results and Discussion

### Initial Batch Investigations

Model substrates to represent aromatic, cyclic aliphatic, and terminal linear aliphatic alkenes were selected, with studies initiated to successfully reproduce literature conditions^17^ followed by examination of aspects of the methodology that could be varied to make it more amenable to an industrial process.

Previous reports (Figure 1) used a Mn(OTf)_2_ catalyst with PAA_M_ (15 wt%) as the oxidant, but this work demonstrates the application of the less expensive Mn(OAc)_2_ (Table 1) and MnCl_2_ (Table S1) along with a resin-synthesized peracetic acid (PAA_R_) (15 wt%) for epoxidation of the model substrates.^17^Table 1 shows the conditions developed in batch, with methanol required when preparing stock solutions of Mn(OAc)_2_ due to the poor solubility of Mn(OAc)_2_ in acetonitrile.

**Table 1:**
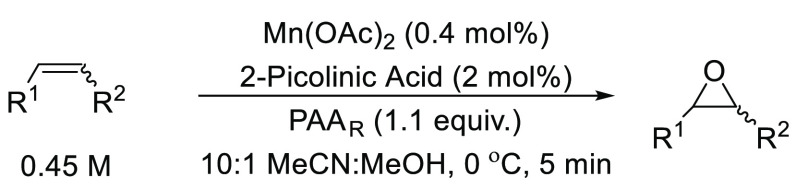

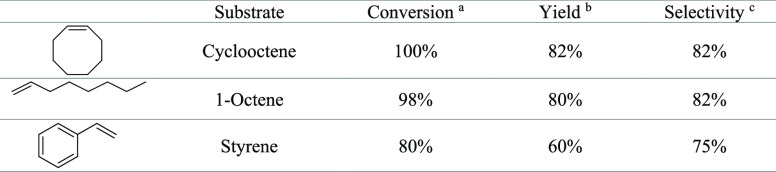
Batch Studies of the Epoxidation of Alkene Substrates with PAA_R_ and Mn(OAc)_2_^a^

a Reactions were carried out at a 1 mmol (substrate) scale in 2 mL of a 10 mM solution of 2-picolinic acid in acetonitrile with 0.2 mL of a 20 mM solution of Mn(OAc)_2_ in methanol. PAA_R_ (15 wt%) synthesized in flow (0.55 mL) was added at a rate of 50 μL/10 s at 0 °C.

b Conversions and yields were determined by GC-FID using benzonitrile as an internal standard.

c Selectivity is defined as the percentage of reacted starting material converted to the corresponding epoxide.

Initial studies found that the reactions were indeed very fast, proceeding to >80% conversion in under 5 min, and therefore well-suited to the development of a continuous flow system. The thermal properties of this reaction were investigated by calorimetric studies (see the Supporting Information (SI) for further details), which indicated that for the reaction to be safely scaled in semibatch conditions, the addition of peracetic acid would have to be at a low and controlled rate. This slow addition is required to ensure that the reaction is controllable at scale and to avoid a potential adiabatic temperature rise of 60.5 °C. Therefore, a flow approach could allow for the development of a more efficient and safer process. The reactivity of epoxides, which renders them valuable intermediates, also makes their preparation challenging due to the formation of other products, including the corresponding diol, keto alcohol, and aldehyde.^31,32^ Nonetheless, the selectivity for the desired products for this catalytic method compares well to those for alternative systems.^12,33,34^ Analysis of these reaction mixtures was carried out to determine whether there were any major byproducts which could be identified. In the case of styrene, one major byproduct, phenylacetaldehyde (formed *via* Meinwald rearrangement of the epoxide^35^) was formed in 15% yield (confirmed by ^1^H NMR and GC-MS), in agreement with results reported by Stack and co-workers.^17^ This brings the mass balance for this reaction to 95%. As mentioned, a range of products can be formed in oxidation systems, and in this case, benzaldehyde was observed (by ^1^H NMR and GC-MS analysis). In the case of the aliphatic substrates, 1-octene and cyclooctene, there was no one significant byproduct that could be observed, with no GC peaks with area greater than 1% of the epoxide product (see the SI for GC traces). Difficulty in identifying all byproducts is common in such catalytic epoxidation studies.^34,36−39^

### Peracetic Acid Synthesis in Flow

An acidic polymer resin was utilized for the catalytic synthesis of PAA, as this is a proven method and is well-suited to flow systems.^28,40^ A number of different commercially available polymeric sulfonic acid resins were tested, and all were found to produce peracetic acid solutions of similar concentration (Table 2); there was also little variation in results for the epoxidation reaction compared to the base-modified solution employed by Stack and co-workers^17^ (Table 3). Amberlyst 36, a macroporous sulfonic acid resin, was selected for further study. The experimental setup employed (Table 2) consisted of a packed bed of acidic polymer resin within an Omnifit borosilicate glass column (Omnifit SolventPlus chromatography column with one fixed and one adjustable endpiece, 10 mm × 400 mm), and a mixture of acetic acid and hydrogen peroxide was pumped through. The column was packed with acidic resin, adjusting the column to a volume of 25.5 mL, and the void volume was calculated to be 2.85 mL based on a residence time of 19 min for a 0.15 mL/min flow rate (calculated from the time taken for a solution to pass through a column of dry resin).

**Table 2:**
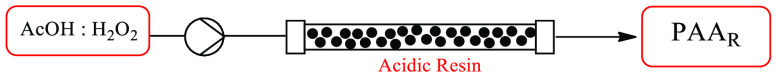
Variation of Resin in Peracetic Acid Synthesis

entry	resin	wt% PAA^c^	yield (%)^d^
1^a^	Amberlite IR120 (H^+^ form)	16	95
2^a^	Amberlyst 16 (H^+^ form)	16	95
3^a^	Amberlyst 15 (H^+^ form)	15	90
4^a^	Amberlyst 36 (H^+^ form)	16	95
5^b^	Amberlyst 36 (H^+^ form)	15	76

a Using 85:15 v/v AcOH/H_2_O_2_ (aq. 50 wt%) as the feed solution with a flow rate of 0.5 mL/min.

b Using 7:3 v/v AcOH/H_2_O_2_ (aq. 30 wt%) as the feed solution with a flow rate of 0.15 mL/min.

c Determined by titration with potassium permanganate followed by an iodometric titration as outlined in the SI.

d Yield with respect to H_2_O_2_.

**Table 3:**
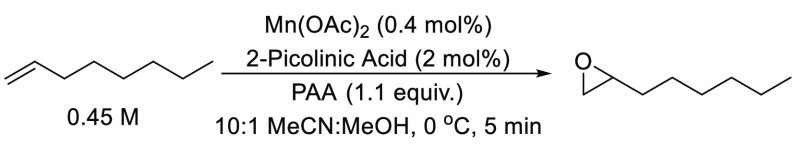
Variation of Peracetic Acid Solution Used in Batch Epoxidation of 1-Octene

PAA used	conversion (%)^d^	yield (%)^d^
PAA_M_^a^	100	80
PAA_R50_^a^^,^^b^	100	81
PAA_R30_^a^^,^^b^	99	78
PAA_R30_^b^^,^^c^	80	60

a PAA added at 50 μL/10 s.

b PAA_R50_ = PAA_R_ synthesized using 50 wt % H_2_O_2_; PAA_R30_ = PAA_R_ synthesized using 30 wt% H_2_O_2_.

c PAA was added in one aliquot.

d Conversions and yields were determined by GC-FID using benzonitrile as an internal standard.

The residence time required to produce the desired 15 wt% peracetic acid concentration is dependent on the concentration of hydrogen peroxide used, and shorter residence times can be used if 50 wt% H_2_O_2_(aq) is employed rather than 30 wt% (Table 2). To further enhance the process safety, the more dilute solution was chosen for development work. These reactions were conducted at room temperature; as shown by other studies, higher temperatures would enable shorter residence times.^28,41,42^

### Catalytic Flow Studies

**Figure 2:**
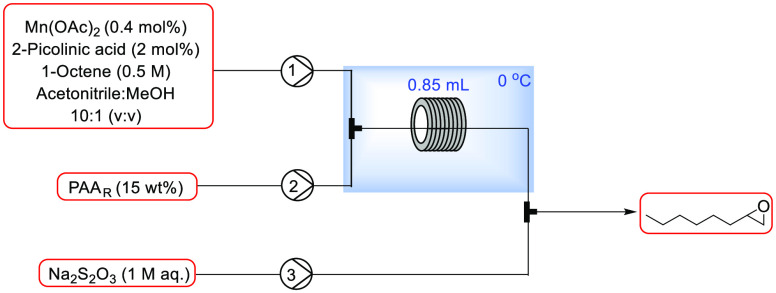
Initial setup for flow reactions with Mn(OAc)_2_.

Initially, a system with three pumps was employed: one pump introduced PAA_R_ (preprepared), a second pump delivered a stock solution of substrate and catalyst, and a third pump was used to continuously add an aqueous solution of sodium thiosulfate as a quench stream (Figure 2). The equipment used included SF10 Vaportec peristaltic pumps with blue peristaltic tubing (which demonstrated the best compatibility with the reaction components) and PTFE tubing (1/16″ o.d., 0.5 mm i.d.) for the reactor coil and additional loops for precooling/premixing. It was important to use a metal-free setup in order to avoid metal-catalyzed decomposition of the peracid. As indicated in the schematics, the precooling loop and reactor tubing were cooled in an IPA/water bath using a Huber immersion chiller, whereas all of the other components were at ambient temperature. When using the flow system with *in situ* PAA synthesis a back-pressure regulator (Zaiput Flow Technologies, cat. no. BPR-10) was used.

Using the setup shown in Figure 2, a precipitate was found to develop over time in the feed solution (channel 1), and this led to a blockage in the reactor tubing. This precipitate originated from Mn(OAc)_2_, as it occurred in the absence of substrate and was not an issue when Mn(OTf)_2_ was used. It is known that the acetate ion can act as a bridging ligand to form Mn(II) coordination polymers, which is a plausible explanation for this insoluble manganese species.^43^ When reactions are carried out in flow, the handling and formation of solids are well-known challenges, and researchers have tackled this *via* sonication, increased temperature, periodic washes, or dilution.^44,45^ Flushing the reactor system with water successfully cleared the tube blockages, but intermittent washing was not a feasible option in this case due to the frequency of clogging. The addition of water to the solvent system was investigated, but a 4:1 v/v acetonitrile:water ratio was required to prevent precipitation, and this led to a reduced yield of epoxide (Table S2). To address this solubility issue, a five-pump setup was used, in which the Mn(OAc)_2_, 2-picolinic acid, substrate, and peracetic acid were introduced as different feeds. (Table 4). The precipitating species takes time to accumulate; by performing the metal ligation in flow, precipitation is prevented because the metal complex is formed *in situ* and quickly enters the reactor coil, where it then contacts the PAA solution, and there is no observed precipitation.

**Table 4:**
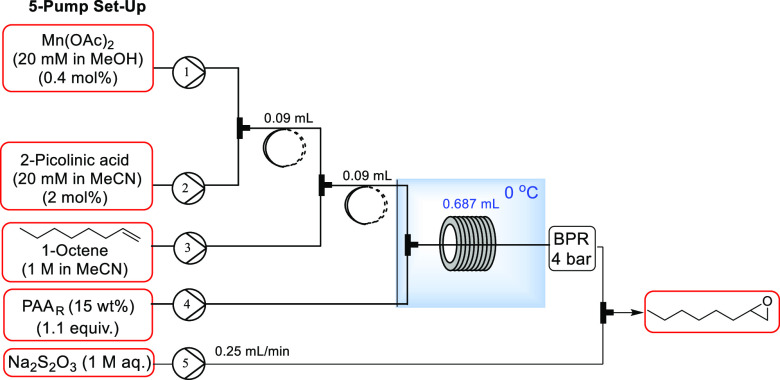
Flow Setup with *In Situ* Metal Ligation and Variation of the Conditions

*T* (°C)	residence time (s)	equiv of PAA	conv. (%)^a^	yield (%)^a^
0	30^b^	1.1	84	63
17	30^b^	1.1	85	60
0	60^c^	1.1	85	59
0	25^d^	2.2	100	53

a Conversions and yields were determined by GC-FID using benzonitrile as an internal standard.

b The flow rates of pumps 1, 2, 3, and 4 were 0.1, 0.5, 0.5, and 0.275 mL/min, respectively.

c The flow rates of pumps 1, 2, 3, and 4 were 0.05, 0.25, 0.25, and 0.138 mL/min, respectively.

d The flow rates of pumps 1, 2, 3, and 4 were 0.1, 0.5, 0.5, and 0.550 mL/min, respectively.

This setup was successful in eliminating the issue of reactor fouling, allowing for run times of 8 h without any back-pressure increase or precipitation observed. Under these conditions, 85% conversion of 1-octene with a 63% yield of 1,2-epoxyoctane could be achieved. This yield was lower than had been obtained in initial batch studies (Table 1). Development studies were carried out with the aim of achieving results comparable to those observed in batch. However, varying the residence time and the temperature had little impact, and the yields remained significantly lower than those obtained in batch (Table 4).

In the batch procedure, PAA is slowly added over 2 min, as addition of a single aliquot negatively affects results (Table 3). This highlights the impact of the oxidant concentration. A competing metal-catalyzed decomposition of PAA that reduces the amount of oxidant available to carry out the epoxidation is a possible explanation. Titration of a control reaction mixture (Mn(OAc)_2_ and 2-picolinic acid) with no substrate found that 80% of the PAA had decomposed after 5 min. Simply increasing the amount of PAA achieves full conversion for 1-octene, but this is detrimental to the epoxide selectivity. This is likely due to subsequent reaction of the epoxide.^31,32^

To replicate the gradual addition of PAA used in batch, the effect of introducing PAA *via* three separate streams was assessed, as shown in Table 5. In this case, Mn(OTf)_2_ was used, as this allowed the use of a catalyst/substrate stock solution without the problems of precipitation. The addition of PAA_R_ across numerous points in the reaction tubing was studied, but the epoxide yield did not improve (Table 5).

**Table 5:**
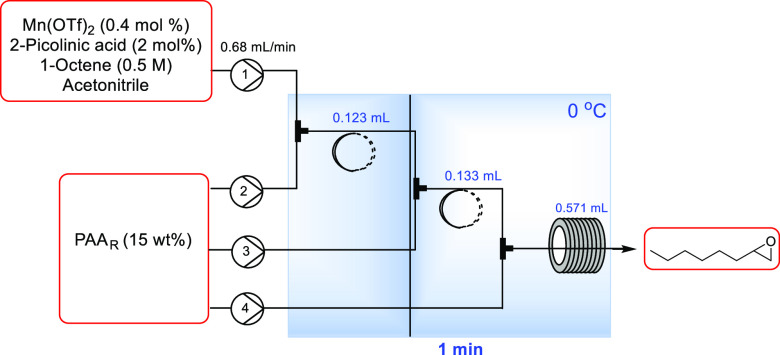
Results from PAA Split Addition

equiv of PAA	split ratio	conv. (%)^a^	yield (%)^a^
1.2	no split^b^	85	63
1.2	1:1:1^b^	83	61
1.2	2:1:1^b^	80	60
1.2	4:1:1^b^	83	61
2	no split^c^	99	53
2	1:1:1^c^	99	55

a Determined by GC with an internal standard (benzonitrile).

b The sum of flow rates 2, 3, and 4 was 0.176 mL/min.

c The sum of flow rates 2, 3, and 4 was 0.293 mL/min.

As can be seen in Table 5, 1-octene undergoes 83% conversion with 61% epoxide yield when the PAA is split across three different points of the reactor. Although the yields obtained remained lower than previously observed in batch, this is potentially due to high local concentrations as a consequence of the rate of addition of peracetic acid. Following these results, it was decided to explore catalyst speciation, as this can also have a significant impact on the performance of the reaction.

### Increasing the Ligand:Mn Ratio

In solution, an equilibrium between several Mn(II)/2-picolinic acid species is expected, and it was previously predicted that a Mn(OTf)_2_ to 2-picolinic acid ratio of 1:5 would maximize the concentration of bisligated species (see the SI of ref (18)). The ratios of PAA to Mn and ligand are important and clearly have an impact on catalyst speciation, which is key not only for the epoxidation pathway but also in controlling PAA decomposition.^46−48^ Control reactions carried out in batch (Table 6) demonstrated that increasing the ligand:metal ratio mitigates the impact of higher PAA concentrations. Addition of PAA in one aliquot when using a 5:1 ligand:metal ratio results in an 18% decrease in conversion (see entry 1 vs entry 2 in Table 6), while using a 20:1 ligand to metal ratio allows for PAA addition in a single aliquot without a negative effect on the results (see entry 3 vs entry 4 in Table 6).

**Table 6:** Batch Control Experiments for Ligand Variation with Single Aliquot PAA Addition

entry	Mn(OAc)_2_ loading (mol %)	ligand:Mn	mode of PAA addition	conv. (%)^a^	yield (%)^a^
1	0.4	5:1	one aliquot	80	60
2	0.4	5:1	over 2 min	98	80
3	0.05	20:1	one aliquot	95	80
4	0.05	20:1	over 2 min	98	78

a Conversions and yields were determined by GC-FID using benzonitrile as an internal standard.

A range of ligand:metal ratios were assessed to improve performance under continuous flow conditions. As shown in Table 7, the influence of the ligand:metal ratio was assessed, as were the effects of temperature and residence time. To eliminate any potential effect of precipitation on the stoichiometry, Mn(OTf)_2_ was selected for this study. It was demonstrated that a Mn(OTf)_2_ loading of just 0.05 mol% with a 20:1 ligand:Mn ratio at 0 °C could match the performance previously achieved in batch studies (see Table 1). These results further illustrate the importance of the PAA to Mn and ligand ratios. The conditions outlined in entry 3 were found to be the most attractive, with lower temperatures and longer residence times adding little benefit.

**Table 7:**
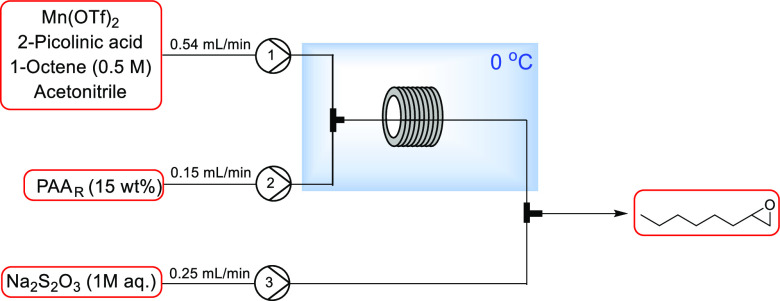
Screening of Ligand:Mn Ratios and Temperatures

entry	Mn(OTf)_2_ loading (mol %)	ligand:Mn	*T* (°C)	residence time (min)	conv. (%)^a^	yield (%)^a^
1	0.05	10:1	0	7^b^	79	68
2	0.05	10:1	–10	7^b^	82	73
3	0.05	20:1	0	5^c^	100	78
4	0.05	20:1	–10	7^b^	82	73
5	0.1	10:1	0	3^c^	95	73
6	0.1	10:1	–10	7^b^	98	72
7	0.1	20:1	0	3^c^	98	83
8	0.1	20:1	–10	7^b^	100	72
9	0.01	10:1	0	1^d^	16	14
10	0.05	30:1	0	1^d^	86	74
11	0.4	10:1	0	1^d^	95	73

a Determined by GC with an internal standard (benzonitrile).

b Reactor volume of 4.7 mL.

c Reactor volume of 2 mL.

d Reactor volume of 0.74 mL.

Having demonstrated performance comparable to batch results by varying the ligand:metal ratio, the continuous flow Mn(OAc)_2_-catalyzed epoxidation with *in situ* synthesis of PAA was demonstrated (Table 8). To incorporate the peracetic acid synthesis, the peracetic acid output from the packed column was fed directly to the reactor tube, mixing with the substrate and catalyst at a T-piece.

**Table 8:**
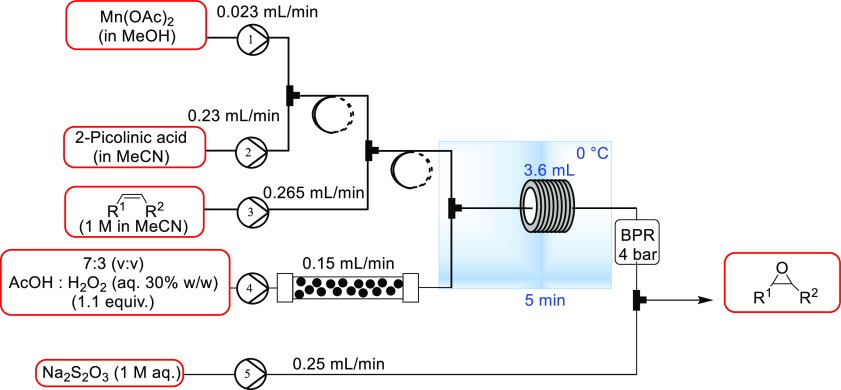
Flow System Developed for Alkene Epoxidation with Mn(OAc)_2_

substrate	Mn(OAc)_2_ loading (mol%)	2-picolinic acid loading (mol%)	conv. (%)^a^	yield (%)^a^
cyclooctene^b^	0.05	1	100	83 (76^d^)
1-octene^b^	0.05	1	100	78^e^
styrene^b^	0.05	1	32	26
styrene^c^	0.2	4	93	63

a Determined by GC with an internal standard (benzonitrile, which is in the substrate feed).

b 5.5 mM Mn(OAc)_2_ solution in MeOH and 11 mM 2-picolinic acid solution in MeCN.

c 22 mM Mn(OAc)_2_ solution in MeOH and 44 mM 2-picolinic acid solution in MeCN.

d Isolated yield.

e Consistently obtained over 8 h of operation.

These conditions were also applied to cyclooctene and styrene, and the results are shown in Table 8. Styrene required a higher catalyst loading to achieve yields comparable to those obtained in batch under these conditions, but the loading is still comparatively low. To demonstrate the scalability and robustness of this flow procedure, the process was run for 8 h with 1-octene. The reaction proceeded for 8 h without any blockages, and sampling at 30 min intervals showed that 100% conversion with 78% epoxide yield was maintained throughout, delivering 16.32 g of 1,2-epoxyoctane at a rate of 2.04 g/h.

Control experiments further demonstrated the effectiveness of this catalytic method. Rutjes and co-workers previously reported a study on the continuous flow dihydroxylation of cyclohexene (*via* epoxidation).^49^ In that study it was reported that oxidation with peracetic acid could be carried out without a catalyst at elevated temperatures (*e.g.*, 60 °C). The results of this study found that good yields of epoxide could be obtained by heating without the Mn catalyst for cyclooctene (Table S4), but this thermal approach was not applicable for styrene and 1-octene (Table S5). The Mn(II)/2-picolinic acid method is attractive for synthesis because it has previously been shown to have a wide substrate scope,^17^ and this work has demonstrated that it can be operated continuously in a scalable manner.

## Conclusions

A continuous flow method for the epoxidation of alkenes has been developed using a Mn(II)/2-picolinic acid catalyst with peracetic acid as the oxidant. In flow, varying the ligand:metal ratio proved to be beneficial. Epoxide yields of up to 83% could be obtained with loadings of Mn(OAc)_2_ as low as 0.05 mol% and 2-picolinic acid at 1 mol%. This continuous flow system is an alternative for process chemists to have in their toolbox when considering carrying out the epoxidation of alkenes on a larger scale. The protocol reported herein avoids the precipitation of solids, allowing long-term operation. Future work on the scale-up of such a flow process could be achieved by using established approaches, for example, scale-out using a numbering-up strategy with multiple reactors or scale-up by increasing the size of the reactor^50,51^ Numbering-up maintains the benefits of using small-diameter tubing, whereas larger reactor tubing would likely require the use of mixing elements to maintain the efficiency of mixing and heat exchange.^52^

## Supporting Information Available

Greater detail on the equipment used, including a photo of the flow setup; formulae used to calculate reagent stoichiometry, conversion, and yield; additional reaction data related to the effect of varying the Mn(II) salt, the solvent mix, and the ligand:Mn ratio; calorimetry data; full experimental detail and characterization data for the isolation of cyclooctene oxide; and sample GC spectra for reactions (PDF)

## Abbreviations

AcOH: acetic acid
BPR: back-pressure regulator
EtOAc: ethyl acetate
GC-FID: gas chromatography with flame ionization detector
HDPE: high-density polyethylene
MeCN: acetonitrile
MeOH: methanol
PAA: peracetic acid
PAA_M_: base-modified commercial peracetic acid (10:3:13 v/v/v 32% PAA/10% KOH(aq)/AcOH)
PAA_R_: resin-synthesized peracetic acid
