# Supplementary material for: Continuous Flow Epoxidation of Alkenes Using a Homogeneous Manganese Catalyst with Peracetic Acid
Source: Org Process Res Dev. 2023 Jan 14;27(2):262–8. doi: 10.1021/acs.oprd.2c00222 (PMC9942194; doi:10.1021/acs.oprd.2c00222)
Supplement: Supplementary file 1 — op2c00222_si_001.pdf [file op2c00222_si_001.pdf]

Continuous Flow Epoxidation of Alkenes Using a Homogeneous Manganese Catalyst with Peracetic Acid

Supporting Information

Ailbhe A. Ryan[a,b,c], *Seán D. Dempsey*[a,b,c], *Megan Smyth*[a], *Karen Fahey*[b], *Thomas S. Moody*[a,b] *Scott Wharry*[a], *Paul Dingwall*[c], *David W. Rooney*[c], *Jillian M. Thompson*[c*], *Peter C. Knipe*[c*], *and Mark J. Muldoon*[c*]

[a]Almac Group, Craigavon, BT63 5QD, United Kingdom

[b]Arran Chemical Company, Roscommon, N37 DN24, Ireland

[c] Queen's University Belfast, BT9 5AG, United Kingdom

* jillian.thompson@qub.ac.uk
* p.knipe@qub.ac.uk
* m.j.muldoon@qub.ac.uk

Contents

Experimental 3

Determination of Substrate Conversion and Epoxide Yield using GC-FID 4

Methods for catalytic batch experiments 5

Methods for Flow Experiments 6

PAA_R Synthesis 6

Analysis of Peracetic Acid by Titration 7

Epoxidation Reactions in Flow 8

Isolation of Cyclooctene Oxide 9

Calculations for Catalyst Loadings and Peracetic Acid Equivalents 10

Additional Reaction Data 12

Calorimetric Study 12

Variation in Mn(II) salt used 13

Acetonitrile: Water Batch Studies 14

Conditions Screening for Styrene 14

High Temperature Catalyst Free System 15

Example GC-Traces 16

NMR Spectra of Cyclooctene Oxide 17

References 18

Experimental
General Remarks

Unless otherwise stated, all reagents used were purchased from commercial suppliers and were used without further purification. *cis*-cyclooctene (Sigma Aldrich, 95%), cyclooctene oxide (Sigma Aldrich, 99%), 1-octene (Sigma Aldrich, 98%), 1,2-epoxyoctane (Sigma Aldrich, 96%), styrene (Sigma Aldrich, 99%), styrene oxide (Sigma Aldrich, 97%), manganese acetate (Sigma Aldrich, 98%), manganese bis(trifluoromethanesulfonate) (Sigma Aldrich), manganese chloride (Sigma Aldrich), manganese perchlorate hexahydrate (98%, Sigma Aldrich), 2-picolinic acid (Alfa Aesar, 99%), hydrogen peroxide in water (50 wt.% in H_2O, stabilized, Sigma Aldrich), hydrogen peroxide in water (30 wt.% in H_2O stabilized, Sigma Aldrich), peracetic acid (PAA) (~32 wt%, Sigma Aldrich).

Caution: At the end of reactions, solutions should be quenched using a suitable reagent, such as sodium thiosulfate. It is particularly important, that prior to any concentrating of solutions (*e.g.* in order to isolate a product), the presence of peroxides should be determined using peroxide test strips. If peroxides are present, they should be removed using a suitable reducing agent.

^{1}H NMR spectra were recorded on a Bruker AVX400 (400 MHz) spectrometer at ambient temperature. ^{13}C NMR spectra were recorded on a Bruker AVX400 (101 MHz) spectrometer at ambient temperature. Gas chromatography analysis was carried out using Agilent 7820-A series gas chromatograph with flame-ionization detection (FID).

A Zebron ZB-5 column (30 meter x 320 µm x 0.25 µm) was employed for all the separations. For all methods the helium flow rate was 0.63 mL/min, the injector temperature was 300 °C, the

FID temperature was 300 °C with a hydrogen/air mix for effluent combustion and an injection volume of 1 μL was used. For GC analysis, the method used was as follows: after 1 min at 50 °C, the temperature was increased by 15 °C/min to 70 °C. After 2 min at 70 °C, the temperature was increased by 25 °C/min to 200 °C. The temperature was increased by 40 °C/min to 320 °C.

Determination of Substrate Conversion and Epoxide Yield using GC-FID

The GC yield of products and conversion of substrates were determined using benzonitrile as internal standard, which was found to be stable/unreactive under the reaction conditions. The relative response factor (RF) of analytes was determined by analyzing known quantities of internal standard against known quantities of substrate and product, for cyclooctene, styrene and 1-octene reactions the epoxide products were commercially available. The relative response factor (RF) of analytes was determined by analyzing known quantities of internal standard (benzonitrile) against known quantities of substrate and product according to the following equation:

$$\mathrm{RF} = \frac{\mathrm{Area}_{\mathrm{internal\ standard}} \times \mathrm{Moles}_{\mathrm{analyte}}}{\mathrm{Area}_{\mathrm{analyte}} \times \mathrm{Moles}_{\mathrm{internal\ standard}}}$$

The quantity of an analyte was then calculated according to the following equation:

$$\mathrm{Moles}_{\mathrm{Analyte}} = \frac{\mathrm{RF} \times \mathrm{Moles}_{\mathrm{internal\ standard}} \times \mathrm{Area}_{\mathrm{Analyte}}}{\mathrm{Area}_{\mathrm{internal\ standard}}}$$

$$\mathrm{Epoxide\ Selectivity} = 100 * \left(\frac{\mathrm{mol\ of\ epoxide}}{\mathrm{mol\ of\ reacted\ substrate}}\right)$$

Methods for catalytic batch experiments

General Notes

Acetonitrile was used as solvent for stock solutions of $Mn(OTf)_2$ and $Mn(ClO_4)_2$, while methanol was used for experiments with $Mn(OAc)_2$ and $MnCl_2$.

PAA_M is a modified solution of commercial peracetic acid (PAA_C) (32 wt.% (aq.)). PAA_M was made up fresh on the day of use, commercial peracetic acid (2 mL 38-40 wt.% (aq.)) was neutralized with 10% aqueous KOH (0.6 mL), then diluted to a 1:1 v/v mixture with AcOH (2.6 mL) at room temperature. If larger volumes of the solution are to be made the addition of 10% KOH to PAA should be carried out at 0 °C to mitigate the exotherm.

For experiments using resin synthesized PAA (PAA_R), no modification was required, and it was used directly after the resin had been filtered out or after collection from the packed column.

General Procedure for Oxidation of Alkenes (adapted from the method reported by Stack and co-workers[1])

To a 15 mL glass sample vial fitted with a Teflon coated magnetic stirrer bar, Mn(II) salt (0.2 mL of 20 mM solution, 4 µmol, 0.4 mol%) and 2-picolinic acid (2 mL of 10 mM acetonitrile solution, 20 µmol, 2 mol %) were added. Substrate (1 mmol) and benzonitrile internal standard (0.7 mmol) were added. This solution was stirred at 0 °C. PAA_M (0.55 mL, 1.1 mmol, 1.1 equiv.) was added in 50 µL aliquots at 10 s intervals. The reaction was stirred at 0 °C for 5 mins, inclusive of PAA_M addition. Samples for GC analysis were passed through a plug of basic aluminum oxide to remove metal-containing species and diluted using ethyl acetate.

Methods for Flow Experiments
Flow Equipment

Vapourtec SF-10 peristaltic pumps, with the "blue tubing" (for solvent compatibility) were used.[2] Reactor tubing was PTFE (1/16" OD, 0.5 mm ID). An Omnifit borosilicate glass column (Diba Omnifit® SolventPlus™ Chromatography Column w/ 1 Fixed & 1 Adjustable Endpiece, 10 x 400 mm) was used for the packed bed of acidic resin. A Zaiput BPR-10 was the back pressure regulator used. A Huber TC45E immersion chiller was used to control the temperature of the IPA/water bath. The decomposition of peracetic acid and hydrogen peroxide can be readily catalyzed by metals, therefore all fittings and tubing used were made of perfluorinated polymers (PTFE tubing and ETFE tee-pieces).[3,4] Peristaltic pumps were employed which ensures that reagents are not exposed to metal components (this also applied to the synthesis of PAA as shown in Table 2 of the manuscript).

PAA_R Synthesis
Batch Preparation of Peracetic Acid

The method was based on literature precedents[5] and was as follows: In a round-bottomed flask acetic acid (15 mL), H_2O_2 (50 wt.% (aq.), 5 mL, 73.5 mmol) was stirred with acidic resin (2 g) at 27 °C overnight. This was then filtered through a plug of cottonwool to remove the resin.

In-situ Peracetic Acid Synthesis for Flow

A length of 32 cm (25 mL) of an Omnifit glass column was packed with sulfonic acid polymer resin and subsequently washed with acetic acid. A solution of hydrogen peroxide and acetic acid was mixed in a high-density polyethylene (HDPE) bottle. This was pumped through the packed column.

To establish the residence time of liquid in the column, a liquid displacement study was performed, it was found that at 0.5 mL/min it took 19 minutes to pass methanol through the column. This was used as a reference residence time and collection of PAA for analysis began after 3 x residence time to allow for steady state to be reached. For experiments using pre-synthesized PAA_R, the solution was collected directly from the column without any modification and stored in a HDPE bottle.

Analysis of Peracetic Acid by Titration [6,7]

The sample of peracid was accurately weighed and placed in a 500 mL conical flask containing 100 mL of 5 % sulfuric acid and sufficient ice to maintain a temperature of between 0 °C and 10 °C. To determine the hydrogen peroxide content of the peracid, it was titrated against a 0.01 M solution of potassium permanganate, standardized against a standard solution of sodium oxalate. The end point was identified when the solution turned from colorless to a light pink. At this point, 10 mL of 10 % potassium iodide solution was added to the flask, the liberated iodine causes the solution to turn red brown. To determine the peracetic acid content, it is then titrated with 0.1 M sodium thiosulfate, when approaching the endpoint 3 drops of starch indicator was added, causing the solution to turn dark blue/brown. Titration was continued until the solution turned from dark blue to colorless. An average of two titres was taken in each case (ensuring they were ± 0.2 mL).

Calculations:

$$\%\ H_2O_2 = \frac{\text{mL. of potassium permanganate} \times M \times 85.05}{10 \times \text{sample weight (g)}}$$

$$\%\ \text{Peracetic acid} = \frac{\text{mL. sodium thiosulfate} \times M \times 38.03}{10 \times \text{sample weight (g)}}$$

Where M = the molarity of the titrant

Epoxidation Reactions in Flow

Continuous Flow Experiments with 3-Pumps

The continuous flow set-up depicted in Figure 2 was utilized. The set up consisted of three streams; the first stream was a solution of $Mn(OAc)_2$, 2-picolinic acid, substrate, and internal standard in acetonitrile/MeOH (10:1 v/v). The second stream was pre-synthesized PAA_R. Stream 1 and 2 combined at a tee-piece and went through 0.5 mm ID reactor tubing. The third stream was an aqueous 1 M sodium thiosulfate solution and was introduced at the end of the reaction to quench any remaining peroxide species. Before commencing any sampling, the system was run for adequate time to allow for three reactor volumes to ensure steady state.

Continuous Flow Experiments with Split Addition of PAA

The continuous flow set-up depicted in Table 5 was utilized. The set up consisted of four streams; the first stream was a solution of $Mn(OTf)_2$, 2-picolinic acid, substrate and benzonitrile internal standard in acetonitrile. The three other streams introduced pre-synthesized PAA_R at different points along the course of the reaction *via* tee-pieces.

Continuous Flow Experiments with 5-Pumps

The continuous flow set-up depicted in Table 4 in the manuscript was utilized. The set up consisted of five reagent streams The first stream was a 5.5 mM solution of $Mn(OAc)_2$ in methanol. The second reagent stream was an 11 mM solution of 2-picolinic acid in acetonitrile; it was combined with the $Mn(OAc)_2$ solution *via* a tee-piece. A 1 M solution of substrate and internal standard in acetonitrile was then introduced at a tee-piece. The catalyst solution and the substrate

solution were passed through a coil of tubing to allow for mixing to occur before combining with pre-synthesized PAA_R at a tee-piece. An aqueous 1 M sodium thiosulfate solution was introduced at a tee-piece. Before commencing any sampling, the system was run for 60 minutes to ensure steady state.

We also used a 5-pump system where PAA is prepared *in-situ* and this is illustrated in Table 8 in the manuscript and below in Figure S1.

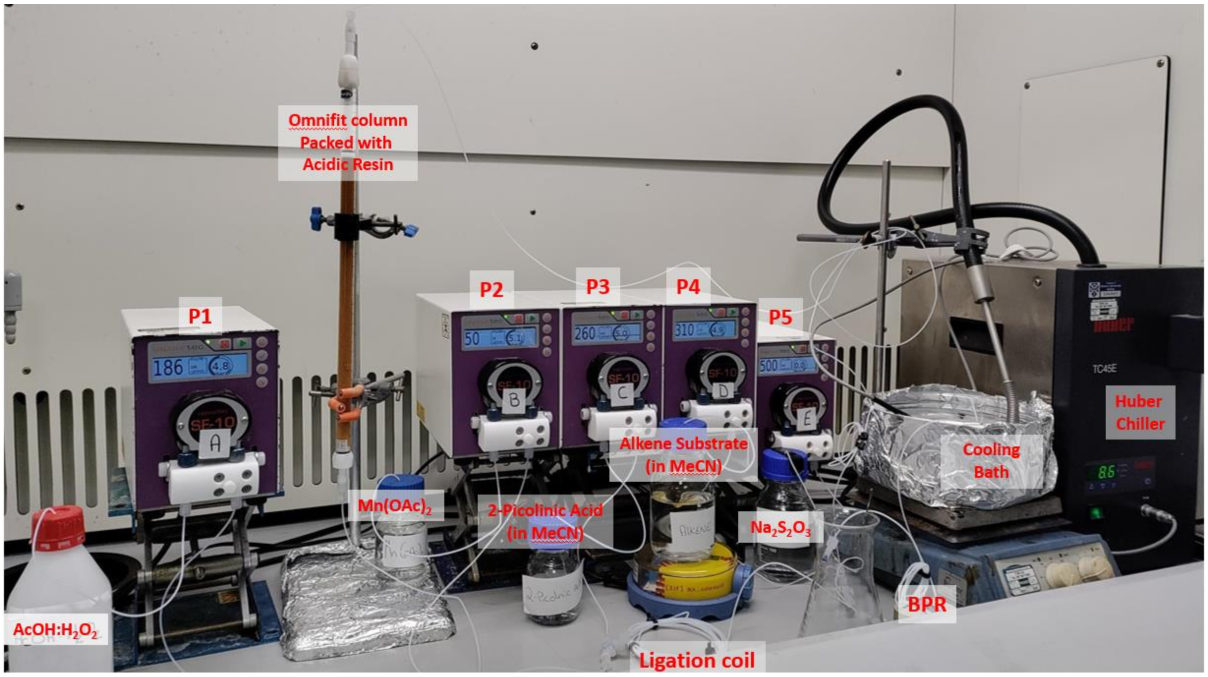

Figure S1 Photograph of 5-pump set-up for alkene epoxidation with $Mn(OAc)_2$

Isolation of Cyclooctene Oxide

As outlined earlier, the conversion and yields were determined by GC analysis, which is most appropriate for such volatile model substrates. It was also demonstrated that a product (cyclooctene oxide) could be isolated, using a similar approach to previously described by Stack and co-workers for a range of substrates.[1]

Upon exiting the flow reactor, the reaction mixture (which has been quenched in-line) was collected for one hour (equates to 1.75 g cyclooctene, 0.0159 mol). The sample was checked for remaining peroxides using peroxide test strips. The solution was then diluted with CH_2Cl_2 (50 mL). This mixture was then poured into a saturated solution of $NaHCO_3$ (50 mL). After separation of the layers, the aqueous layer was extracted with CH_2Cl_2 (2 x 50 mL). The organic extract was washed with a saturated solution of $NaHCO_3$ (50 mL) to remove any residual AcOH, and the aqueous phase was again extracted with CH_2Cl_2 (50 mL). The combined organic extracts were dried over $MgSO_4$, filtered, and concentrated by rotary evaporation. The product was purified by column chromatography on a silica gel stationary phase with 2% EtOAc in pentane as the mobile phase (Rf~ 0.3) to afford cyclooctene oxide as a colourless solid (1.49g, 74 % yield). ^{1}H NMR (400 MHz, $CDCl_3$) δ 3.01 – 2.77 (m, 2H), 2.26 – 1.97 (m, 2H), 1.69 – 1.22 (m, 10H). ^{13}C NMR (101 MHz, $CDCl_3$) δ 55.66, 26.55, 26.28, 25.59. Data are in agreement with those reported in the literature.[8]

Calculations for Catalyst Loadings and Peracetic Acid Equivalents

Φ- flow rate (mL/min); C- Molar concentration

Molar ratio of A to B: $= \frac{\Phi_A \cdot C_A}{\Phi_B \cdot C_B}$

$$\text{PAA Equivalents} = \frac{\Phi_{PAA} \cdot C_{PAA}}{\Phi_{Substrate} \cdot C_{Substrate}}$$

$$\text{Mol \% of Mn} = \frac{\Phi_{Mn(II)} \cdot C_{Mn(II)}}{\Phi_{Substrate} \cdot C_{Substrate}} * 100$$

$$\text{Mol\% of 2-picolinic acid} = \frac{\Phi_{2-picolinic\ acid} \cdot C_{2-picolinic\ acid}}{\Phi_{Substrate} \cdot C_{Substrate}} * 100$$

Example Calculation for Optimized Conditions;

Pump A: Φ_1 =0.15 mL/min C_1= 2 M PAA; $\Phi_A . C_A$= 0.3 mmol

Pump B: Φ_2= 0.023 mL/min C_2= 0.005.5 M $Mn(OAc)_2$; $\Phi_B . C_B$ = 0.0001265 mmol

Pump C: Φ_3= 0.230 mL/min C_3= 0.011 M 2-picolinic acid $\Phi_C . C_C$= 0.00253 mmol

Pump D: Φ_4= 0.265 mL/min C_4= 1 M 1-octene; $\Phi_D . C_D$ = 0.265 mmol

$Mn(OAc)_2$ Loading= (0.0001265 mmol/0.265 mmol)*100 ~0.05 mol %

2-Picolinic Acid Loading= (0.00253 mmol/0.265 mmol)*100 ~1 mol %

PAA Equivalents= 0.3 mmol/ 0.265 mmol ~ 1.1 equiv

Additional Reaction Data

Calorimetric Study

Manganese(II) acetate (0.076g) in methanol (22.0 mL), 2-picolinic acid (0.271g) in acetonitrile (220 mL), 1-octene (12.33 g) and benzonitrile (10 mL) were charged to a 250 mL Atlas calorimeter. The temperature was adjusted to 5 °C and a Heat Flow Calorimetry experiment was carried out. After calibration a 2 M solution of PAA_R (61 mL) was added *via* a dropping funnel over 19 min. After the addition was complete, the reaction was held for an additional 17 min before quenching with 1 M sodium thiosulphate in water (90 mL) over 9 min. The reaction was held for 29 min before calibration (time 28 min). The mixture was then discharged.

The results are shown below:

Process	Exp. scale	ATR	Exotherm class.	Heat of reaction*			Power exp.*				P @ 1 kg		P @ 10 kg		Accu. approx.
							Maximum		Average		Max	Av.	Max	Av.	
	[g]	[°C]		[kJ]	[kJ/mol]	[kJ/kg]	W	W/kg	W	W/kg	W	W	kW	kW	[%]
Reaction	12.33	60.5	Medium	36.9	335.8	133.1	44.2	159.4	32.4	116.8	3585	2628	38.9	26.3	0
Quench	12.33	16.2	Low	16.0	145.6	42.0	30.9	81.1	29.6	77.7	2506	2401	25.1	24.0	<5%

* Kg here reflects total reaction mass.

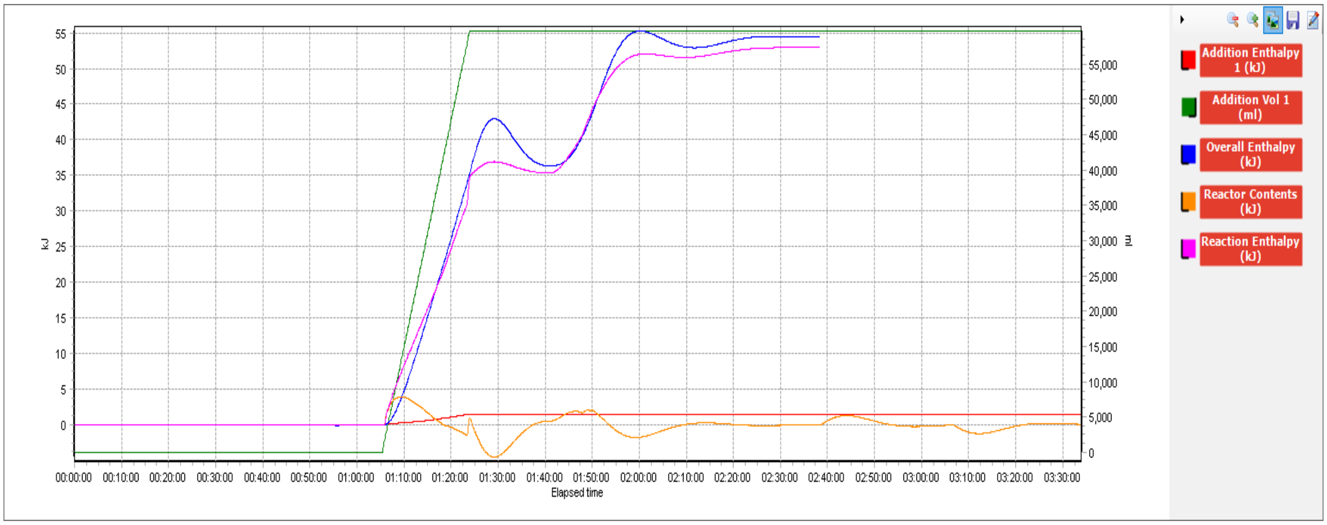

From the Calorimetry Data:

- The observed heat of reaction was 36.9 kJ, 335.8 kJ mol^{-1} or 133.1 kJ kg^{-1} of total reaction mass.

- The heat capacity (C_P) of the starting reaction mixture was calculated to be 2.2 $Jg^{-1}K^{-1}$. The C_P of the end reaction mixture was 2.2 $Jg^{-1}K^{-1}$.
- The Adiabatic Temperature Rise (ATR) was 60.5 °C and the Maximum Temperature Synthesis Reaction (MTSR) was 65.5 °C. Starting from 5 °C the batch will just reach reflux.
- There was no accumulation in this reaction.

Variation in Mn(II) salt used

Table S1 Results from epoxidation of model alkene substrates with variation of Mn(II) salt in batch

Substrate	Mn(II)	Conversion	Yield
1-Octene	$Mn(OTf)_2$	100 %	80 %
	$Mn(ClO_4)_2.6H_2O$	100 %	79 %
	$MnCl_2$	99 %	79 %
	$Mn(OAc)_2$	98 %	78 %
Cyclooctene	$Mn(OTf)_2$	100 %	78 %
	$Mn(ClO_4)_2.6H_2O$	100 %	78 %
	$MnCl_2$	99 %	82 %
	$Mn(OAc)_2$	98 %	82 %
Styrene[a]	$Mn(OTf)_2$	81 %	63 %
	$Mn(ClO_4)_2.6H_2O$	83 %	60 %
	$MnCl_2$	84 %	62 %
	$Mn(OAc)_2$	84 %	62 %
[a] Major by-product identified as phenylacetaldehyde by NMR and GC-MS			

Acetonitrile: Water Batch Studies

Table S2: Influence of water on product yield

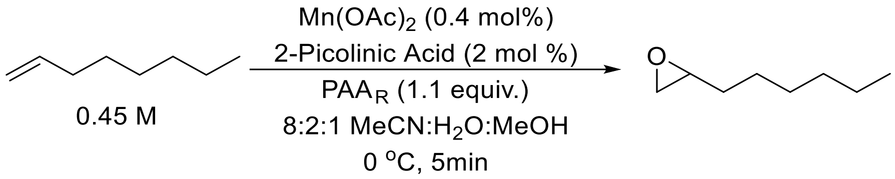

Substrate	Conversion	Yield
1-Octene	95 %	74 %
Cyclooctene	94 %	78 %
Styrene	52 %	36 %

Conditions Screening for Styrene

Table S3 Variation of conditions for styrene

$Mn(OTf)_2$	Ligand: Mn	Temperature	Conversion	Yield
0.1 mol%	10:1	0 ºC	42 %	28 %
0.1 mol%	10:1	15 ºC	42 %	27 %
0.1 mol%	20:1	0 ºC	66 %	43%
0.2 mol%	10:1	0 ºC	65 %	42 %
0.2 mol%	10:1	15 ºC	74 %	48 %
0.2 mol%	20:1	0 ºC	93 %	62 %
0.2 mol%	20:1	15 ºC	87 %	49 %
0.3 mol%	20:1	0 ºC	73 %	43 %
0.4 mol%	20:1	0 ºC	82 %	50 %
0.4 mol%	20:1	-10 ºC	95 %	60 %

High Temperature Catalyst Free System

Table S4: Results obtained at higher temperatures without the use of a catalyst for cyclooctene

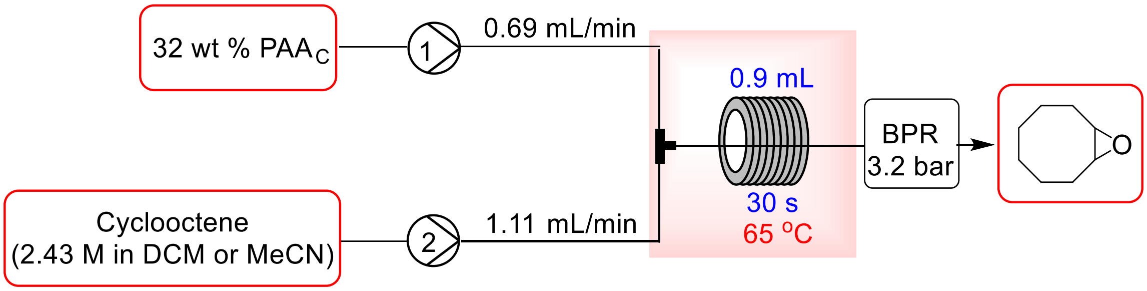

Solvent	PAA	Conversion	Yield
Dichloromethane	PAA_C	99 %	88 %
Acetonitrile	PAA_C	100 %	78 %
Acetonitrile	PAA_R	87 %	79 %

Table S5: Results obtained at higher temperatures without the use of a catalyst for other substrates

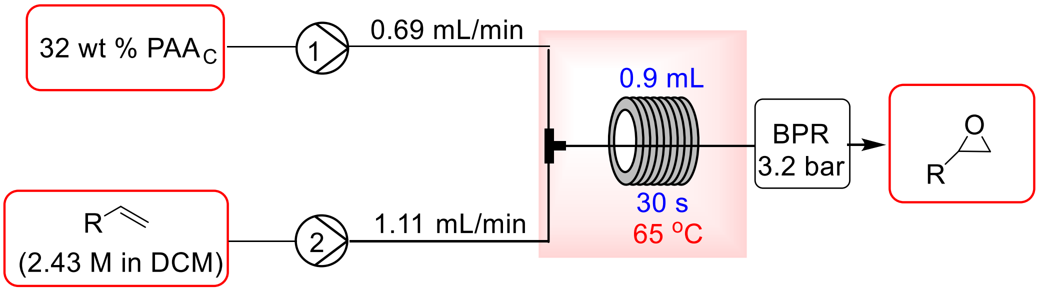

Substrate	PAA	Conversion	Yield
1-Octene	PAA_C	17 %	7 %
Styrene	PAA_C	23 %	8 %

Example GC-Traces

Styrene Reaction Mixture

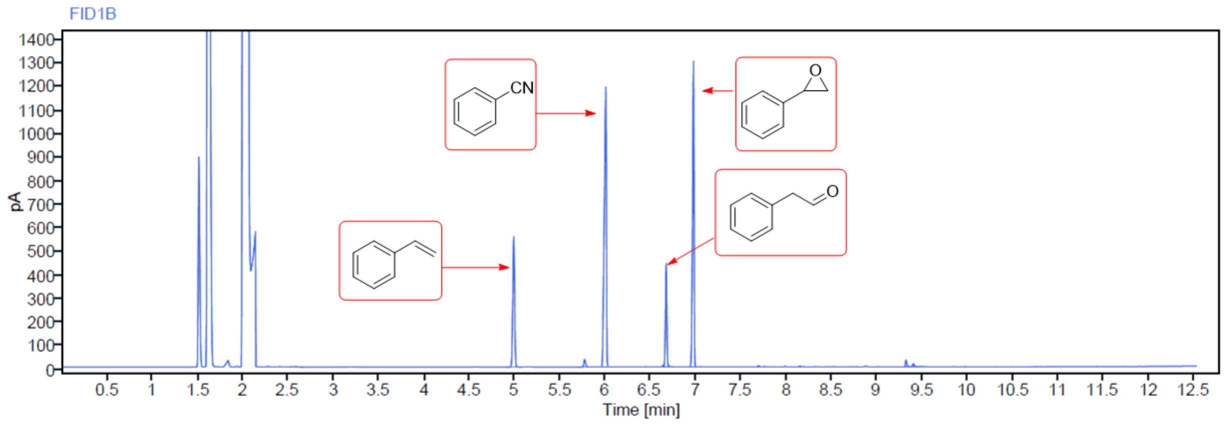

1-Octene Reaction Mixture

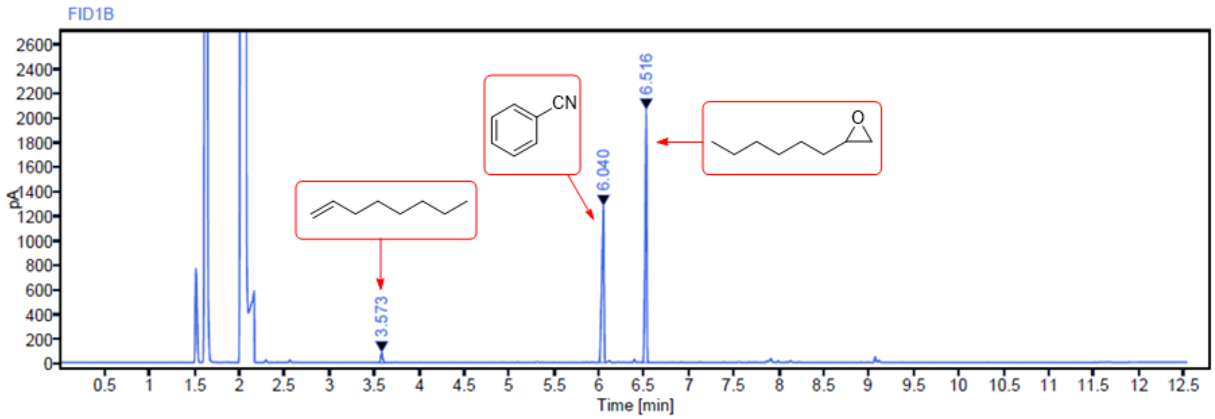

Cyclooctene Reaction Mixture

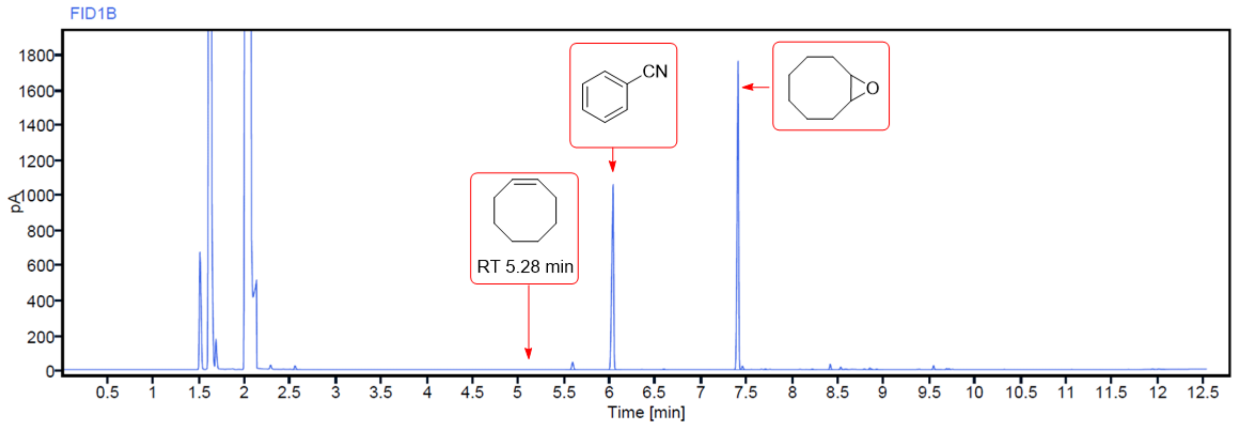

NMR Spectra of Cyclooctene Oxide

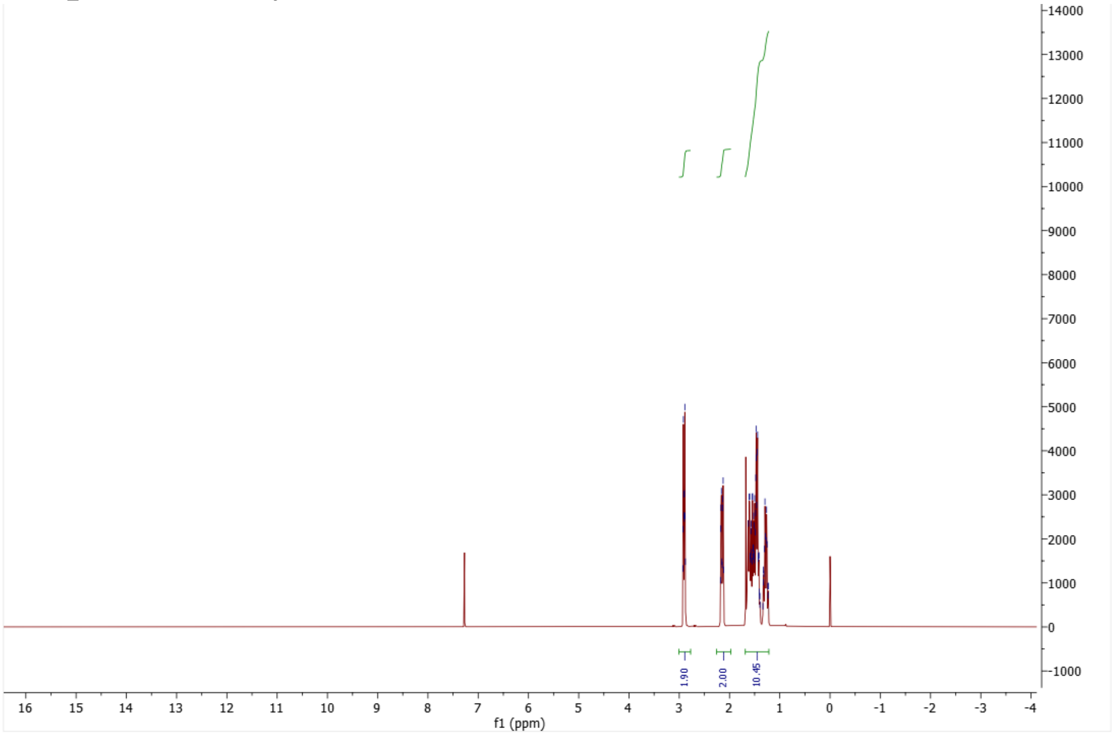

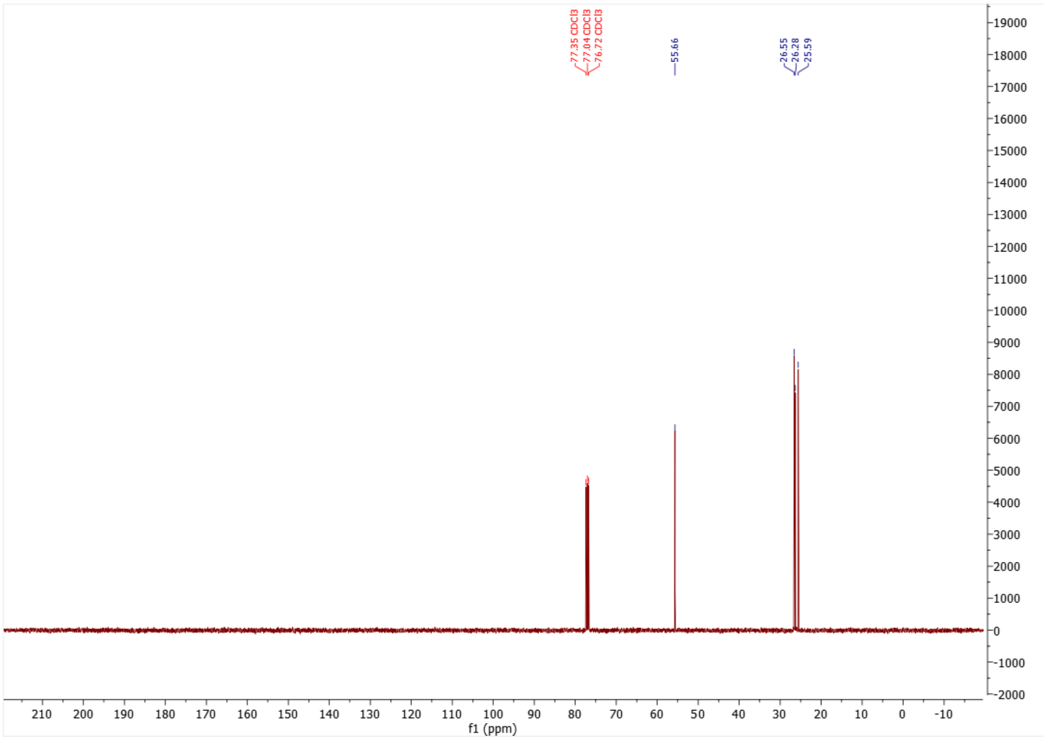

References

(1) Moretti, R. A.; Du Bois, J.; Stack, T. D. P. Manganese(II)/Picolinic Acid Catalyst System for Epoxidation of Olefins. *Org. Lett.* **2016**, *18* (11), 2528–2531. https://doi.org/10.1021/acs.orglett.6b00518.

(2) Vapourtec.com https://www.vapourtec.com/products/sf-10-pump-features/ (accessed January 2023).

(3) Popov, E.; Eloranta, J.; Hietapelto, V.; Vuorenpalo, V. M.; Aksela, R.; Jäkärä, J. Mechanism of Decomposition of Peracetic Acid by Manganese Ions and Diethylenetriaminepentaacetic Acid (DTPA). *Holzforschung* **2005**, *59* (5), 507–513. https://doi.org/10.1515/HF.2005.084.

(4) Yuan, Z.; Ni, Y.; Van Heiningen, A. R. P. Kinetics of the Peracetic Acid Decomposition: Part II: pH Effect and Alkaline Hydrolysis. *Can. J. Chem. Eng.* **1997**, *75* (1), 42–47. https://doi.org/10.1002/cjce.5450750109.

(5) Murphy, A.; Pace, A.; Stack, T. D. P. Ligand and pH Influence on Manganese-Mediated Peracetic Acid Epoxidation of Terminal Olefins. *Org. Lett.* **2004**, *6* (18), 3119–3122. https://doi.org/10.1021/ol048846l.

(6) Greenspan, F. P.; MacKellar, D. G. Analysis of Aliphatic Per Acids. *Anal.Chem.* **1948**, *20* (11), 1061–1063.

(7) D'Ans, J.; Frey, W. Untersuchungen Über Die Bildung von Persäuren Aus Organischen Säuren Und Hydroperoxyd. *Zeitschrift für Anorg. Chemie* **1913**, *84* (1), 145–164. https://doi.org/10.1002/zaac.19130840114.

(8) Dong, J. J.; Saisaha, P.; Meinds, T. G.; Alsters, P. L.; Ijpeij, E. G.; van Summeren, R. P.; Mao, B.; Fañanás-Mastral, M.; de Boer, J. W.; Hage, R.; et al. Oxidation of Alkenes with H_2O_2 by an In-Situ Prepared Mn(II)/Pyridine-2-Carboxylic Acid Catalyst and the Role of Ketones in Activating H_2O_2. *ACS Catal.* **2012**, *2* (6), 1087–1096. https://doi.org/10.1021/cs3002226.
